# Developing an infection prevention and control intervention to reduce hospital-acquired infections in Cambodia and Lao People’s Democratic Republic: the HAI-PC study protocol

**DOI:** 10.3389/fpubh.2023.1239228

**Published:** 2023-09-20

**Authors:** Sreymom Oy, Chan Hang Saing, Mengieng Ung, Marina Zahari, Inthavong Nouhak, Sothea Kim, Michiko Nagashima-Hayashi, Dyna Khuon, Virya Koy, Sovatha Mam, Somphou Sayasone, Vonthanak Saphonn, Siyan Yi

**Affiliations:** ^1^Saw Swee Hock School of Public Health, National University of Singapore and National University Health System, Singapore, Singapore; ^2^Department of International Program for Health in the Tropics, Lao Tropical and Public Health Institute, Vientiane, Lao People’s Democratic Republic; ^3^University of Health Sciences, Phnom Penh, Cambodia; ^4^Department of Hospital Services, Ministry of Health, Phnom Penh, Cambodia; ^5^KHANA Center for Population Health Research, Phnom Penh, Cambodia; ^6^Public Health Program, College of Education and Health Sciences, Touro University California, Vallejo, CA, United States

**Keywords:** disinfection practices, hand hygiene, healthcare workers, infection control, behavior changes, low- and middle-income countries (LMICs)

## Abstract

**Background:**

Hospital-acquired infections (HAIs) are significant public health issues, especially in low-and middle-income countries (LMICs). Hand hygiene and low-level disinfection of equipment practices among healthcare workers are some of the essential measures to reduce HAIs. Various infection prevention and control (IPC) interventions to reduce HAI incidence have been developed. However, effective interventions have not been well developed in the LMICs context. Therefore, this protocol aims to develop, pilot, and assess the feasibility and acceptability of an IPC intervention in Cambodia and the Lao People’s Democratic Republic.

**Methods:**

This study will consist of four phases guided by the Medical Research Council (MRC) Framework. Three hospitals will be purposely selected – each from the district, provincial, and national levels – in each country. The gap analysis will be conducted in Phase 1 to explore IPC practices among healthcare workers at each hospital through desk reviews, direct observation of hand hygiene and low-level disinfection of equipment practices, in-depth interviews with healthcare workers, and key informant interviews with stakeholders. In Phase 2, an IPC intervention will be developed based on the results of Phase 1 and interventions selected from a systematic literature review of IPC interventions in LMICs. In Phase 3, the developed intervention will be piloted in the hospitals chosen in Phase 1. In Phase 4, the feasibility and acceptability of the developed intervention will be assessed among healthcare workers and representatives at the selected hospitals. National consultative workshops in both countries will be conducted to validate the developed intervention with the national technical working groups.

**Discussion:**

The MRC Framework will be employed to develop and evaluate an intervention to reduce HAIs in two LMICs. This theoretical framework will be used to explore the factors influencing hand hygiene compliance among healthcare workers. The gap analysis results will allow us to develop a comprehensive IPC intervention to reduce HAI incidence in Cambodia and Lao People’s Democratic Republic. Findings from this protocol will feed into promising IPC interventions to reduce HAI incidence in other resource-limited settings.

**Clinical trial registration:**

ClinicalTrial.Gov, identifier NCT05547373.

## Introduction

Infection prevention and control (IPC) has been recognized as a vital component in health systems as it affects patients and healthcare workers (1). Inadequate adherence to the standard protocols of IPC results in hospital-acquired infections (HAIs) that often occur in patients in medical care (2). HAIs have received little public health attention, particularly among policymakers in low-and middle-income countries (LMICs) (3). As a result, millions of patients are affected by HAIs each year globally. A wide gap exists between countries in the HAI prevalence, varying between 3.5–12% in high-income countries and 5.7–19.1% in LMICs at any given time. WHO further recognizes a lack of evidence on IPC in LMICs, suggesting that more research in these countries is warranted.

The recent coronavirus disease 2019 (COVID-19) pandemic has proven the significance of IPC in saving lives by reducing HAIs. The stringent and enhanced IPC measures introduced at various hospitals to fight against the pandemic have reduced HAI incidence markedly (4–8). However, the prevalence of HAIs could be extremely high in LMICs. For instance, the HAI prevalence among adults in intensive care units in Vietnam was estimated to be 29.5% in 2015 (9). Regarding the economic burden of HAIs in LMICs, available United States and Europe data suggest that HAIs cost several billion dollars annually (10). It has been estimated that the five significant HAIs – central line-associated bloodstream infections, ventilator-associated pneumonia, surgical site infections, *clostridium difficile* infections, and catheter-associated urinary tract infection – cost the United States approximately USD 9.8 billion annually (11). Evidence of mortality and increased antibiotic resistance due to HAIs have also been documented (12, 13).

Against this backdrop, several IPC interventions to reduce HAIs have been developed, implemented, and evaluated worldwide (14). In magnet-designated hospitals in California, the United States, effective and adequate implementation of IPC programs, including healthcare workers’ adherence to hand hygiene guidelines, routine fomite disinfection, environmental decontamination, and antimicrobial stewardship programs, have significantly contributed to reducing the HAI incidence (15). A recent study revealed that interventions on structural inputs (e.g., availability of hand sanitizers), standard care processes, and their combination reduced HAI incidence significantly (14). Additionally, the review highlighted promising interventions, including using disinfection technology, such as ultraviolet light and hydrogen peroxide vapor, while noting that many existing studies lack rigor in their design and methods.

Hand hygiene is one of the simplest and most effective measures for preventing HAIs. Hand hygiene prevents patient infections, the hospital environment contamination with pathogens, and the cross-transmission of microorganisms between patients (16). Despite the effectiveness, a study on IPC practices in Cambodia identified barriers to promoting hand hygiene, including inadequate environmental hygiene, insufficient protective equipment, understaffing, overcrowding, and poor knowledge of infection control measures among healthcare workers and caregivers (17). Inadequate staff capacity and commitment, limited support from decision-makers, and financial shortages for IPC implementation have also been iterated (18).

Disinfection of reusable medical devices is another critical component of IPC interventions. Medical equipment that comes into physical contact with patients and healthcare workers is susceptible to colonized pathogenic microorganisms (19). For instance, a study found that stethoscopes and sphygmomanometers were associated with HAIs due to their contamination with bacteria (20). Disinfection of the equipment removes the microorganisms, preventing the transmission of organisms between patients (21). Guidelines for reusable equipment, environmental cleaning, and disinfection are included in the IPC national guidelines in Cambodia and Lao People’s Democratic Republic (22, 23) and WHO’s guidelines on core components of IPC programs at the national and acute healthcare facility levels (2).

Cambodia and Lao People’s Democratic Republic have been striving to tackle HAIs despite inadequate IPC infrastructures, a lack of IPC support services and surveillance and reporting systems for HAI incidence, limited IPC implementation capacity, and financial constraints (24, 25). Moreover, data on HAI prevalence and IPC-related knowledge, attitude, and practice among healthcare workers in LMICs are scarce. Such a paucity of data further prevents researchers from estimating the burden of the HAI endemic and devising proper IPC interventions to reduce HAI incidence.

The national guidelines and training curriculums have been distributed to the subnational levels in Cambodia and Lao People’s Democratic Republic. However, the evaluation mechanisms for IPC implementations and HAI surveillance systems have not been formalized. Based on anecdotal evidence in Cambodia, there has been a strong emphasis on the need for behavioral change among healthcare workers in adhering to the national IPC guidelines and evidence-based research, which is imperative for addressing this challenge. Although it lacks rigor in the methods, a study of hand hygiene education among healthcare workers in a provincial referral hospital in Cambodia showed that hand hygiene education using the WHO Guidelines on Hand Hygiene (26) reduced surgical site infections significantly (24). Therefore, documenting gaps in IPC implementation and practices in the local context to identify the appropriate and feasible IPC intervention components related to behavioral factors is a critical first step to reducing HAIs using evidence-based interventions.

Designing an effective IPC intervention involves several components and varies across healthcare settings. The Medical Research Council (MRC) Framework for Developing and Evaluating Complex Interventions has been employed across many disciplines (27–30). According to this framework (31, 32), the first step in developing a complex intervention is to identify the burden of the problems and the limitations of the current implementations and practices (32). The second step involves identifying the existing interventions. The third step is to develop a theoretical intervention model based on the gap in implementation and practices and the current evidence on effective interventions. Once the intervention is developed, a pilot study and the feasibility and acceptability assessment of the piloted intervention are required.

Based on this framework, this protocol aims to develop, pilot, and assess the feasibility and acceptability of an IPC intervention in Cambodia and Lao People’s Democratic Republic. The protocol’s specific objectives are to (1) conduct a gap analysis of IPC implementation and practices among healthcare workers at district, provincial, and national hospitals to identify IPC intervention components, (2) develop an IPC intervention based on the gap analysis findings and model interventions from a systematic review of IPC interventions to reduce the HAI incidence in LMICs, and (3) pilot the IPC intervention and assess its feasibility and acceptability among healthcare workers and stakeholders in Cambodia and Lao People’s Democratic Republic.

## Methods and analysis

### Study settings and design

The healthcare systems in Cambodia and Lao People’s Democratic Republic are organized into distinct levels, encompassing the district, provincial, and national tiers (33, 34). This study will be conducted in three hospitals in each country, with each hospital purposively selected from each healthcare system level (national, provincial, and district levels). One national hospital will be selected from the country’s capital city. Then, one province will be selected, and the provincial hospital and one district referral hospital will be selected. The intensive care unit, surgery unit, and medical ward will be selected for national and provincial hospitals, and the surgery unit, maternal and child health ward, and medical ward will be selected for district hospitals.

**Figure 1:**
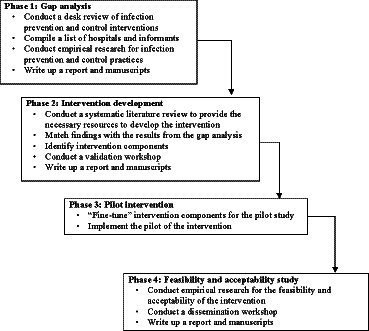
Project milestones and timelines.

The study will be conducted between January 2023 and December 2024. As shown in Figure 1, the IPC intervention development will involve four phases: (1) gap analysis, (2) intervention development, (3) pilot implementation of the developed intervention, and (4) feasibility and acceptability assessment of the pilot implementation (32, 35).

#### Phase 1: Gap analyses

The gap analysis aims to identify gaps in the implementation and practices of hand hygiene and low-level disinfection of equipment guidelines among healthcare workers in the selected hospitals in each country. The gap analysis will involve a desk review of IPC national policies and guidelines, key informant interviews (KIIs) with key stakeholders, in-depth interviews (IDIs) with healthcare workers, and direct hand hygiene and low-level disinfection of equipment practice observations among healthcare workers in the selected hospitals.

Firstly, the “National Guidelines for Infection Prevention and Control in Healthcare Facilities” in Cambodia and Lao People’s Democratic Republic (22, 23) will be reviewed to assess if these guidelines are aligned with the WHO’s “Guidelines on Core Components of Infection Prevention and Control Programs at the National and Acute Health Care Facility Level” (2). Additionally, the policies and guidelines regarding IPC practices during the COVID-19 pandemic will be reviewed.

Secondly, the perspective of key stakeholders from each country on the gaps in IPC implementation and practices among healthcare workers will be explored by conducting KIIs. Three national stakeholders from each country will be recruited. In Cambodia, the national stakeholders will be the representatives of MoH’s Communicable Disease Control Department and the Department of Hospital Services and the selected national hospital. In Lao People’s Democratic Republic, stakeholders will be representatives of the MoH’s Department of Health Care and Rehabilitation, Communicable Disease Control Department, and the selected national hospital. At the provincial level, one representative of the provincial health department and one representative of the selected provincial hospital will be purpovely selected. Finally, two district key stakeholders will be purposely selected – one from the operational district office and one from the selected district hospital. A representative from the WHO for the KII from each country will also be included.

For IDIs, three healthcare workers (a doctor, a midwife, and a nurse) will be selected from each selected hospital ward, totaling 27 healthcare workers per country (Table 1). In addition, direct observations of hand hygiene and low-level disinfection of equipment practices will be conducted concurrently with the IDIs and KIIs at the selected hospitals, targeting doctors, nurses, and midwives. Eligible participants will include healthcare workers who (i) are aged 18 years or older, (ii) have worked in the selected wards of the chosen hospitals for at least 6 months, and (iii) who will be able to and agree to provide informed consent to participate in the study. Interns and visiting healthcare workers will be excluded.

**Table 1:** Sample size across the three hospitals in each country (Cambodia and Lao People’s Democratic Republic).

Health system level	Number of hospitals^a^	Number of IDI participants	Number of KII participants	Total
World Health Organization			1	1
National	1	9	3	12
Provincial	1	9	2	11
District	1	9	2	11
Total	3	27	8	35

IDI, in-depth interview; KII, key-informant interview.

a For the national and provincial hospitals, we will select intensive care units, surgery units, and medical wards. We will select surgery units, maternal and child health wards, and medical wards for district hospitals.

#### Phase 2: A theory-based IPC intervention development

This component aims to identify behavioral change components necessary for developing a behavioral change intervention by pooling the findings obtained in Phase 1 and a systematic review of IPC implementation interventions in LMICs. The intervention development process will consist of the following four components:

##### Identifying behavioral change components

All behavioral change components under the “Theoretical Domains Framework (TDF)” (36) will be listed and components that need improvement will be identified. The TDF domains identified in Phase 1 will be prioritized.

##### Systematic literature review

A systematic literature review will be conducted, providing the necessary resources to develop the intervention for this study. The review aims to identify intervention models used to promote IPC practices among healthcare workers in LMICs. The review protocol will be registered in PROSPERO, an international database of prospectively registered systematic reviews (37). Peer-reviewed studies and grey literature, reporting IPC interventions among healthcare workers and patients in LMICs, published in English will be included. The extracted data will be analyzed and identified common themes or patterns related to intervention models targeting behavioral change in IPC practices among healthcare workers. The detail of the review protocol and findings will be reported elsewhere.

##### Develop intervention components

The behavioral change components identified in Phase 1 will be matched with the intervention components identified in the systematic review. As a guide to design this IPC intervention, the Behavior Change Wheel (BCW) model will be used, which follows eight steps, including defining the problem in behavioral terms, selecting the target behaviors, specifying the target behaviors, identifying what needs to change and intervention functions, policy categories, behavior change techniques, and the mode of delivery (38).

##### Validate the behavior change intervention

Once the intervention is developed, a national consultative workshop in collaboration with the MoH in Cambodia and Lao People’s Democratic Republic will be conducted to validate the intervention. This workshop aims to summarize the findings from gap analysis, relevant interventions from the systematic review, and the developed interventions. Non-governmaental organizations (NGOs) and key stakeholders working on IPC in each country, representative from district, provincial, and national hospitals will be involved to obtain their feedback on the development of the intervention.

#### Phase 3: Pilot intervention

Once the validation is done, the developed intervention will be piloted at the same three hospitals selected in Phase 1 in each country. The representative from each hospital will be approached to inform them the contents of the pilot intervention. Following that, 15 healthcare workers from each of the three selected wards at the national hospitals, 10 from each of the three selected wards at the provincial hospitals, and seven from each of the three selected wards at the district hospitals will be recruited to participate in the pilot intervention (Table 2).

**Table 2:** Sample of participants participate in the pilot intervention in each country (Cambodia and Lao People’s Democratic Republic).

Health system level	Number of hospitals^a^	Number of healthcare workers per ward	Number of wards	Total sample
National	1	15	3	45
Provincial	1	10	3	30
District	1	7	3	21
Total	3	32	9	96

a For the national and provincial hospitals, we will select intensive care units, surgery units, and medical wards. We will select surgery units, maternal and child health wards, and medical wards for district hospitals.

#### Phase 4: Intervention’s feasibility and acceptability assessment

Subsequently, a post-pilot evaluation will be conducted to assess the feasibility and acceptability and identify barriers and challenges in implementing the developed IPC intervention (32, 35). Additionally, post-pilot direct observation of hand hygiene and low-level disinfection of equipment practices among healthcare workers at the same selected wards in Phase 1 of the selected national, provincial, and district hospitals will also be performed. The assessment will comprise IDIs with healthcare workers and KIIs with representative from each hospital in Cambodia and Lao People’s Democratic Republic. For IDIs, 36 healthcare workers in each country (15 from the national hospitals, 12 from the provincial hospitals, and nine from the district hospitals) will be randomly selected from the pool of 96 healthcare workers participating in the pilot intervention (Phase 3; Table 3).

**Table 3:** Sample of participants for the feasibility and acceptability assessment in each country (Cambodia and Lao People’s Democratic Republic).

Health system level	Number of health facilities^a^	Number of healthcare workers	Number of key informants	Total sample
National	1	15	1	16
Provincial	1	12	1	13
District	1	9	1	10
Total	3	36	3	39

IDI, in-depth interview; KII, key-informant interview.

a For the national and provincial hospitals, we will select intensive care units, surgery units, and medical wards. We will select surgery units, maternal and child health wards, and medical wards for district hospitals.

After documenting the results of the feasibility and acceptability assessment of the pilot intervention, a national dissemination workshop will be conducted in each country. The results of the pilot intervention and the findings from the feasibility and acceptability study will be shared during the workshop. This workshop will be used as a platform to discuss this intervention’s potential strengths and limitations. This can be used for planning future scaling-up purposes. Attendees will include those involved with IPC-related policy including MoH, representative from the provincial health department, and operational district office, NGOs working on IPC, and representative from the district, provincial, and national hospitals in each country.

### Tool development

#### For gap analyses

A standardized WHO’s hand-hygiene observation tool for direct observation (Supplementary material 1) will be adapted for this study (39). The observation form contains two main elements: a header and an observation grid. The header will enable the observers to record the time, date, and location of the selected wards. The observation grid allows observers to record the data to compute the hand hygiene compliance of the healthcare workers.

An observation form will be adapted from an existing study (Supplementary material 2) (40) to observe the low-level disinfection of equipment compliance among healthcare workers. Low-level disinfectants are used to disinfect noncritical items that come into contact with skin. According to the national guidelines on IPC for healthcare facilities, the equipment includes bedpans, toilets, urinals, blood pressure cuffs, electrocardiogram leads, thermometers, stethoscopes, beds, and bedside tables (22, 23).

In Phase 1, IDIs using an interview guide (Supplementary material 3) with healthcare workers will be conducted to understand the factors influencing their hand-hygiene practices. A topic guide from an existing study (41) will be adapted, containing questions derived from 14 theoretical domains to capture multiple-level determinants of health-related behavioral change. Furthermore, healthcare workers will be asked how the COVID-19 pandemic has impacted their hand-hygiene practices.

Additionally, A KII topic guide (Supplementary material 4) will be developed to understand the perspective of key stakeholders about the gap in IPC implementation and practices among healthcare workers in both countries, adapting questions from an existing study (42). Questions on the impact of the COVID-19 pandemic on HAI prevention and control in each country will also be included in the interview guide.

#### For assessing the intervention’s feasibility and acceptability

In Phase 4, hand hygiene and low-level disinfection of equipment observations will be conducted using the same forms developed in Phase 1. Additionally, the topic guide questions (Supplementary material 5) will be adapted from a relevant study (43) to explore the feasibility of the developed intervention and if there are any issues with the intervention and its implementation process. Healthcare workers’ experiences with the developed intervention during the pilot Phase will also be explored to assess the feasibility and acceptability of the developed IPC intervention. Post-pilot KIIs among representative from each hospital who will participate in the pilot phase will be conducted. The interviews aim to explore their perspective on the intervention piloted in Phase 3. The questions (Supplementary material 6) will be adapted from the literature (44).

### Training and data collection procedures

Before the data collection in Phases 1 and 4, the study coordinators will train data collectors in a two-day workshop, followed by another day for study tool pretesting. Data collectors will include staff and graduate students from the University of Health Sciences (UHS) in Cambodia and the Lao Tropical and Public Health Institute (Lao-TPHI) in Lao People’s Democratic Republic. The training will cover direct observations and interview techniques, including confidentiality, privacy, and data quality control.

For hand-hygiene observation, training materials will be adapted from WHO’s training tools (45). The adapted training materials will include PowerPoint presentations and an educational video of healthcare workers performing care in hospital settings. The training will include observation of healthcare workers’ hand-hygiene practices at the selected hospitals. Each observation session will last approximately 20 min (+10 min buffer time, depending on the observed activity) (26). The WHO’s five moments of hand hygiene will be applied: (1) before touching patients or patient surroundings, (2) before a clean/aseptic procedure, (3) after body fluid exposure risk, (4) after touching a patient, and (5) after touching the patient’s surroundings (26). The hand-hygiene actions will be recorded when either handwashing with soap and water or alcohol hand rubbing is observed according to the five moments indicated above. To obtain reliable estimates of the compliance rate, WHO recommends at least 200 opportunities for each ward per observation period (26). The observations will be conducted during the regular working hours of 8: 00 AM–12: 00 PM and 1: 00 PM–5:00 PM.

Similarly, observers will be trained to observe practices among healthcare providers. Healthcare providers will be considered practicing low-level disinfection of equipment if they disinfect non-critical medical equipment (i.e., bedpans, electrocardiogram leads, stethoscopes, blood pressure apparatus, thermometers, pulse oximeters, glucometers, beds, and bedside tables) with approved disinfectants before and after the equipment comes to contact with patients.

Study coordinators will also train data collectors on qualitative data collection techniques using interview guides for IDIs and KIIs. In Phase 1, the IDIs questions will be guided by the TDF (46) to assess factors influencing healthcare workers’ hand-hygiene practices. However, rigidly applying the TDF may result in specific determinants or gaps being overlooked. Therefore, the interviewers will be instructed to be open to any responses from the participants so that the responses will be distinct from the pre-determined TDF domains. Moreover, the interviewers will be trained to use simple languages so that the participants can comprehend the questions correctly.

The hand hygiene and low-level disinfection of equipment observation training in Phase 4 will include the same contents as in Phase 1. Additionally, the study coordinators will train interviewers on conducting IDIs and KIIs, including using neutral wording and probing questions.

Following the training in Phases 1 and 4, the interviewers and observers will spend one morning pretesting the topic guides for IDIs and KIIs and the observation form on hand hygiene and low-level disinfection of equipment in one hospital in the capital city of each country. In the afternoon, all the interviewers and observers will reflect on their experience with the study tools and provide feedback.

Potential participants from each selected hospital will be invited through the heads of the selected hospitals and wards and asked them for signed informed consent. Each interview will take approximately 45–60 min to complete. The interviews will be audiotaped, and notes will be taken during the interviews with the participants’ permission. The samples and sampling procedures will be similar in Cambodia and Lao People’s Democratic Republic.

### Data management

Data on hand hygiene and low-level disinfection of equipment observations on paper forms and subsequently enter them into Microsoft Excel 2016 (Microsoft Corporation, Redmond, WA, United States). The study coordinators will upload the audio-recorded IDI and KII data files into a computer folder. The research team will transcribe the audio files in the respective country’s national language (e.g., Khmer and Lao) and store them in another folder. The team will then translate the transcripts into English and keep them in a separate folder. All documents will be password-protected by the study coordinators. The translated transcripts will be anonymized, and unique identification numbers will be assigned instead of personal identifiers to protect the participant’s privacy and confidentiality.

### Data analyses

#### Qualitative data analyses

The study team will transcribe audio recordings from IDIs and KIIs in Phases 1 and 4 verbatim. The transcriptions will then be translated into English. All qualitative data analyses will be performed using NVivo 12 software. For the IDIs in Phase 1, the analyses will follow three steps – coding, generating specific beliefs/themes, and identifying the relevant domains (47).

##### Coding interview transcripts

Two study team members will independently code the transcripts into the 14 domains of TDF using thematic analyses. They will meet and review their coding and definitions of the codes, accompanied by quotes under each code. If there is a disagreement with the coding, the two coders will discuss it and reach a consensus.

##### Generating specific beliefs/themes

One team member will generate specific beliefs/themes for the coded interview quotes in all TDF domains. The second team member will double-check the accuracy of the specific beliefs/themes generated by the first team member. A specific belief/theme is defined as the participant’s responses underlying a similar theme that suggests a barrier or enabler of hand hygiene compliance (41).

##### Identifying relevant theoretical domains

TDF domains through consensus between the two analysts will be identified, judging them based on three factors (47–49). Firstly, the TDF domains relevant to healthcare workers’ hand hygiene compliance if belief/theme statements appear with high frequencies across the interviews will be considered. Secondly, the TDF domain relevant if conflicting beliefs/themes exist will also be considered. Thirdly, the domains will be consided relevant if there is evidence of strong beliefs/themes that may impact hand hygiene practices among healthcare workers. These three factors will be simultaneously considered during the analyses to determine the theoretical domains.

Similarly, the recorded KIIs in Phase 1 and IDIs and KIIs in Phase 4 will be analyzed following the coding manual for qualitative researchers developed by Johnny Saldaña (50) and thematic analyses introduced by Braun and Clarke (51). The framework approach involves familiarizing with the transcription data, generating initial codes, searching for themes, reviewing themes, defining and naming themes, and producing the report (51).

#### Quantitative data analyses

In Phases 1 and 4, hand hygiene and low-level disinfection of equipment compliance rates will be calculated using the number of times healthcare workers followed the appropriate behaviors divided by the total number of observed opportunities and multiplied by 100. A simple tabulations using Stata 17 (Stata Corporation, Texas, United States) will be conducted to summarize the compliance rate by healthcare worker type (doctors, nurses, or midwives). Student’s *t*-tests of the mean difference in hand hygiene and low-level disinfection of equipment compliance rate in Phase 1 (before the intervention) and Phase 4 (after the intervention) will be performed.

## Discussion

This study aims to develop an IPC intervention to reduce HAI incidence in health facilities using the MRC Framework and assess its implementation feasibility and acceptability in Cambodia and Lao People’s Democratic Republic. Hand hygiene among healthcare workers plays a critical role in reducing HAIs. However, a previous study found that after 6 months of hand-hygiene education training among healthcare workers, only 62.4% of the trainees followed the hand-hygiene guidelines (24). Similarly, a recent study in post-natal care rooms and at home in rural Cambodia found a high frequency of hand-hygiene opportunities; however, compliance with hand-hygiene protocol among all caregivers needed to be improved (17). The study found barriers to promoting hand hygiene among healthcare workers, including inadequate environmental hygienic conditions, insufficient protective equipment, understaffing, overcrowding, and poor knowledge of essential infection control measures (17). To our knowledge, there has yet to be an evaluation mechanism for IPC implementation and HAI surveillance systems formalized in Cambodia and Lao People’s Democratic Republic. Documenting IPC implementation and practice gaps in evidence-based interventions is critical.

This study comprises three main research components. The first component will involve a gap analysis of IPC implementation and practices among healthcare workers in Cambodia and Lao People’s Democratic Republic to identify IPC intervention components. The second component aims to develop an IPC intervention based on findings from the gap analysis and model interventions selected from the most recent systematic review of IPC interventions to reduce HAI incidence in LMICs. The final component involves piloting and assessing the feasibility and acceptability of the IPC intervention developed in Phase 2. Since the IPC intervention covers many features and the implementation and practice of IPC intervention might vary across hospitals, the MRC framework will help guide and assist with the development and evaluation of such complex interventions (35).

Previous empirical research has shown that healthcare workers’ adherence to IPC guidelines to fight against the COVID-19 pandemic has reduced HAI incidence significantly (4–8). The IPC intervention developed in this study will play a critical role in reducing HAI incidence in Cambodia, Lao People’s Democratic Republic, and other LMICs. By employing the MRC Framework, we can develop a theory-and evidence-based intervention applicable and feasible according to the given contexts (32). This study aims to furnish policymakers and healthcare authorities in Cambodia and Lao People’s Democratic Republic with essential evidence, enabling informed decisions regarding the implementation of evidence-based interventions and the initiation of a HAI surveillance system. Such measures hold the potential to enhance patients’ health outcomes significantly. Moreover, the findings of this study will contribute to a broader enhancement of global public health by shedding light on HAI concerns within LMICs. This understanding, in turn, can play a role in fortifying preparedness for future outbreaks.

Several limitations are anticipated regarding the intervention development, pilot study, and acceptability and feasibility assessment. Firstly, direct observation of hand hygiene and low-level disinfection of equipment is subjected to the Hawthorne effect (52). Healthcare workers under observation may behave and practice differently when aware of being observed. However, this effect will diminish because we will conduct the observations for 14 days for each selected ward. Secondly, the observations will only be conducted during regular working hours of 8:00 AM–12:00 PM and 1:00 PM–5:00 PM; therefore, the study findings may not reflect the healthcare workers’ compliance with hand-hygiene and low-level disinfection of equipment practices in the remaining time of their duty. Furthermore, this study will be conducted only in one national, one provincial, and one district hospital in each country; therefore, findings from this study may not be generalized to other health facilities.

## Conclusion

This study will adopt the MRC Framework, a widely recognized guideline for devising and assessing intricate interventions. This approach is poised to yield pivotal insights into formulating a resilient and contextually viable IPC intervention. This intervention holds the potential to effectively curtail the HAI incidence within resource-limited settings, including Cambodia and Lao People’s Democratic Republic. Furthermore, this study’s comprehensive exploration will give researchers and policymakers a deeper understanding of the challenges inherent in implementing IPC interventions within the studied contexts. Doing so will facilitate the identification of feasible strategies to surmount these challenges. Ultimately, the findings of this study will significantly contribute to the accumulating body of evidence essential for policymaker-led efforts to develop efficacious IPC interventions aimed at diminishing HAI occurrences within LMICs.

## Ethics statement

The studies involving humans were approved by the National Ethics Committee for Health Research (NECHR) in Cambodia (No. 118NECHR) and Lao People’s Democratic Republic (No. 081/NECHR, 06/09/2022) and the National University of Singapore Institutional Review Board (NUS-IRB Ref: NUS-IRB-2022-494) approved this study. The studies were conducted in accordance with the local legislation and institutional requirements. The participants provided their written informed consent to participate in this study.

## Author contributions

CS and SY conceptualized and designed the study and wrote the grant proposal to obtain funding. SO, MU, SK, MZ, MN-H, DK, SS, SM, VK, and VS supported the study design and method development and provided technical inputs for the study protocol and research materials. SO, MU, IN, SK, DK, and SS are responsible for training, project implementation, and data collection. SO, CS, MU, and SY drafted the manuscript. All authors contributed to the article and approved the submitted version.

## Funding

The Reckitt Global Hygiene Institute (RGHI) funds the study (RGHI RIN: 2021-008). The funder has no role in the study design, data collection, management and analysis, interpretation of data, and manuscript writing.

## Conflict of interest

The authors declare that the research was conducted in the absence of any commercial or financial relationships that could be construed as a potential conflict of interest.

## Publisher’s note

All claims expressed in this article are solely those of the authors and do not necessarily represent those of their affiliated organizations, or those of the publisher, the editors and the reviewers. Any product that may be evaluated in this article, or claim that may be made by its manufacturer, is not guaranteed or endorsed by the publisher.

## References

[1] World Health Organization. Report on the burden of endemic health care-associated infection worldwide [internet]. Geneva, Switzerland: World Health Organization (2011) Available at: https://apps.who.int/iris/bitstream/handle/10665/80135/9789241501507_eng.pdf.

[2] World Health Organization. Guidelines on core components of infection prevention and control programmes at the national and acute health care facility level [internet] World Health Organization (2016) Available at: https://apps.who.int/iris/handle/10665/251730. 27977095

[3] World Health Organization. (2023). Health care-associated infections [internet]. Fact sheet. Available at: https://www.convatec.ca/media/1286/gpsc_ccisc_fact_sheet_en.pdf.

[4] Jabarpour M Dehghan M Afsharipour G Hajipour Abaee E Mangolian Shahrbabaki P Ahmadinejad M . The impact of COVID-19 outbreak on nosocomial infection rate: a case of Iran. Manilal A, editor. Can J Infect Dis Med Microbiol. (2021) 2021:1–6. doi: 10.1155/2021/6650920 PMC7905999 33680220

[5] Cerulli Irelli E Orlando B Cocchi E Morano A Fattapposta F di Piero V . The potential impact of enhanced hygienic measures during the COVID-19 outbreak on hospital-acquired infections: a pragmatic study in neurological units. J Neurol Sci. (2020) 418:117111. doi: 10.1016/j.jns.2020.117111 32892033 PMC7833504

[6] Ponce-Alonso M Sáez de la Fuente J Rincón-Carlavilla A Moreno-Nunez P Martínez-García L Escudero-Sánchez R . Impact of the coronavirus disease 2019 (COVID-19) pandemic on nosocomial Clostridioides difficile infection. Infect Control Hosp Epidemiol. (2021) 42:406–10. doi: 10.1017/ice.2020.454 32895065 PMC7520631

[7] Losurdo P Paiano L Samardzic N Germani P Bernardi L Borelli M . Impact of lockdown for SARS-CoV-2 (COVID-19) on surgical site infection rates: a monocentric observational cohort study. Updat Surg. (2020) 72:1263–71. doi: 10.1007/s13304-020-00884-6 PMC7488636 32926340

[8] Su C Zhang Z Zhao X Peng H Hong Y Huang L . Changes in prevalence of nosocomial infection pre-and post-COVID-19 pandemic from a tertiary Hospital in China. BMC Infect Dis. (2021) 21:1–7. doi: 10.1186/s12879-021-06396-x 34281515 PMC8289622

[9] Phu VD Wertheim HFL Larsson M Nadjm B Dinh QD Nilsson LE . Burden of hospital acquired infections and antimicrobial use in Vietnamese adult intensive care units. PLoS One. (2016) 11:1–15. doi: 10.1371/journal.pone.0147544 PMC4732823 26824228

[10] Storr J Twyman A Zingg W Damani N Kilpatrick C Reilly J . Core components for effective infection prevention and control programmes: new WHO evidence-based recommendations. Antimicrob Resist Infect Control. (2017) 6:6. doi: 10.1186/s13756-016-0149-9 28078082 PMC5223492

[11] Zimlichman E Henderson D Tamir O Franz C Song P Yamin CK . Health care–associated infections: a meta-analysis of costs and financial impact on the US health care system. JAMA Intern Med. (2013) 173:2039–46. doi: 10.1001/jamainternmed.2013.9763 23999949

[12] Khan HA Baig FK Mehboob R. Nosocomial infections: epidemiology, prevention, control and surveillance. Asian Pac J Trop Biomed. (2017) 7:478–82. doi: 10.1016/j.apjtb.2017.01.019

[13] Centers for Disease Control and Prevention. (2021). HAI data and statistics [internet]. Healthcare-associated infections. Available at: http://www.cdc.gov/HAI/surveillance/index.html.

[14] Maurer NR Hogan TH Walker DM. Hospital-and system-wide interventions for health care-associated infections: a systematic review. Med Care Res Rev. (2020) 78:643–59. doi: 10.1177/1077558720952921 32842879

[15] Caparros AC Wyckoff M. Infection control interventions to improve hospital-acquired infection rates in adult-geriatric patients. J Prev Infect Control. (2020) 6:1–11. doi: 10.36648/2471-9668.6.1.01

[16] Longtin Y Sax H Allegranzi B Schneider F Pittet D. Video in clinical medicine. Hand hygiene. N Engl J Med. (2011) 364:e24. doi: 10.1056/NEJMvcm0903599 21449775

[17] Nalule Y Buxton H Macintyre A Ir P Pors P Samol C . Hand hygiene during the early neonatal period: a mixed-methods observational study in healthcare facilities and households in rural Cambodia. Int J Environ Res Public Health. (2021) 18:1–16. doi: 10.3390/ijerph18094416 PMC8122667 33919264

[18] Sok S Kanal K. P279: infection prevention and control program in developing countries: achievements, challenges and opportunities in Cambodia. Antimicrob Resist Infect Control. (2013) 2:P279. doi: 10.1186/2047-2994-2-S1-P279

[19] Amera Birlie T Amare AT Tassew SF Emiru TD Feleke DG Chanie ES. Nurses’ cleaning practice of non-critical medical equipment in the era of COVID 19: a cross-sectional study in Debre-Tabor comprehensive specialized hospital. Heliyon. (2021) 7:e07626. doi: 10.1016/j.heliyon.2021.e07626 34307954 PMC8288225

[20] Weldegebreal F Admassu D Meaza D Asfaw M. Non-critical healthcare tools as a potential source of healthcare-acquired bacterial infections in eastern Ethiopia: a hospital-based cross-sectional study. SAGE Open Med. (2019) 7:205031211882262–7. doi: 10.1177/2050312118822627 PMC6317151 30693084

[21] World Health Organization. Prevention of hospital-acquired infections: a practical guide. WHO/CDS/CSR/EPH/2002.12. Geneva, Switzerland: World Health Organization (2002).

[22] Ministry of Health. National guidelines for infection prevention and control for healthcare facilities. Phnom Penh: Ministry of Health (2017).

[23] Ministry of Health. National guidelines for infection prevention and control for healthcare facilities in Lao PDR. Vientiane: Ministry of Health (2020).

[24] Sansam S Yamamoto E Srun S Sinath Y Moniborin M Sim KB . Assessment of hand hygiene compliance after hand hygiene education among health care workers in Cambodia. Nagoya J Med Sci. (2016) 78:151–62. 27303102 PMC4885815

[25] Cambodia. Lao People’s Democratic Republic, Myanmar, Viet Nam: greater Mekong subregion health security project. Manila: Asian Development Bank (2016).

[26] World Health Organization & WHO Patient Safety. Hand hygiene technical reference manual: to be used by health-care workers, trainers and observers of hand hygiene practices. Geneva: World Health Organization (2009).

[27] Balaji M Chatterjee S Koschorke M Rangaswamy T Chavan A Dabholkar H . The development of a lay health worker delivered collaborative community based intervention for people with schizophrenia in India. BMC Health Serv Res. (2012) 12:1–12. doi: 10.1186/1472-6963-12-42 22340662 PMC3312863

[28] Bobrow K Farmer A Cishe N Nwagi N Namane M Brennan TP . Using the Medical Research Council framework for development and evaluation of complex interventions in a low resource setting to develop a theory-based treatment support intervention delivered via SMS text message to improve blood pressure control. BMC Health Serv Res. (2018) 18:1–15. doi: 10.1186/s12913-017-2808-9 29361934 PMC5782371

[29] Lakshman R Griffin S Hardeman W Schiff A Kinmonth AL Ong KK. Using the Medical Research Council framework for the development and evaluation of complex interventions in a theory-based infant feeding intervention to prevent childhood obesity: the baby milk intervention and trial. J Obes. (2014) 2014:1–10. doi: 10.1155/2014/646504 PMC4131118 25143830

[30] Paul G Smith SM Whitford D O’Kelly F O’Dowd T. Development of a complex intervention to test the effectiveness of peer support in type 2 diabetes. BMC Health Serv Res. (2007) 7:136. doi: 10.1186/1472-6963-7-136 17764549 PMC2080630

[31] Campbell M Fitzpatrick R Haines A Kinmonth AL Sandercock P Spiegelhalter D . Framework for design and evaluation of complex interventions to improve health. BMJ. (2000) 321:694–6. doi: 10.1136/bmj.321.7262.694 10987780 PMC1118564

[32] Craig P Dieppe P Macintyre S Mitchie S Nazareth I Petticrew M. Developing and evaluating complex interventions: the new Medical Research Council guidance. BMJ. (2008) 337:a1655. doi: 10.1136/bmj.a1655 18824488 PMC2769032

[33] Akkhavong K Paphassarang C Phoxay C Vonglokham M Phommavong C Pholsena S. Lao People’s Democratic Republic health system review, Vol. 4. Geneva, Switzerland: Health Systems in Transition (2014).

[34] Annear PL Grundy J Ir P Jacobs B Men C Nachtnebel M . The kingdom of Cambodia health system review, Vol. 5. Geneva, Switzerland: Health Systems in Transition (2015).

[35] Medical Research Council. A framework for development and evaluation of RCTs for complex interventions to improve health. London: Medical Research Council (2000).

[36] Cane J O’Connor D Michie S. Validation of the theoretical domains framework for use in behaviour change and implementation research. Implement Sci. (2012) 7:37. doi: 10.1186/1748-5908-7-37 22530986 PMC3483008

[37] National Institute for Health and Care Research. (2023). International prospective register of systematic reviews [internet]. Available at: https://www.crd.york.ac.uk/PROSPERO/.

[38] Michie S Atkins L West R. The behaviour change wheel: a guide to designing interventions [internet] Silverback Publishing (2014) Available at: http://www.behaviourchangewheel.com/.

[39] World Health Organization. (2022). Monitoring tools [internet]. Available at: https://www.who.int/teams/integrated-health-services/infection-prevention-control/hand-hygiene/monitoring-tools.

[40] Powell-Jackson T King JJC Makungu C Spieker N Woodd S Risha P . Infection prevention and control compliance in Tanzanian outpatient facilities: a cross-sectional study with implications for the control of COVID-19. Lancet Glob Health. (2020) 8:e780–9. doi: 10.1016/S2214-109X(20)30222-9 32389195 PMC7202838

[41] Squires JE Suh KN Linklater S Bruce N Gartke K Graham ID . Improving physician hand hygiene compliance using behavioural theories: a study protocol. Implement Sci. (2013) 8:1–9. doi: 10.1186/1748-5908-8-16 23379466 PMC3571966

[42] Mugambe RK Mselle JS Ssekamatte T Ntanda M Isunju JB Wafula ST . Impact of mhealth messages and environmental cues on hand hygiene practice among healthcare workers in the greater Kampala metropolitan area, Uganda: study protocol for a cluster randomized trial. BMC Health Serv Res. (2021) 21:88. doi: 10.1186/s12913-021-06082-3 33499864 PMC7835669

[43] Shrestha R Altice FL Didomizio E Sibilio B Ranjit YS Copenhaver MM. Feasibility and acceptability of an mhealth-based approach as an HIV prevention strategy among people who use drugs on pre-exposure prophylaxis. Patient Prefer Adherence. (2020) 14:107–18. doi: 10.2147/PPA.S236794 32021122 PMC6971384

[44] Bowen DJ Kreuter M Spring B Cofta-Woerpel L Linnan L Weiner D . How we design feasibility studies. Am J Prev Med. (2009) 36:452–7. doi: 10.1016/j.amepre.2009.02.002 19362699 PMC2859314

[45] World Health Organization. (2022). Training tools [internet]. Available at: https://www.who.int/teams/integrated-health-services/infection-prevention-control/hand-hygiene/training-tools.

[46] McGowan LJ Powell R French DP. How can use of the theoretical domains framework be optimized in qualitative research? A rapid systematic review. Br J Health Psychol. (2020) 25:677–94. doi: 10.1111/bjhp.12437 32558289

[47] Squires JE Linklater S Grimshaw JM Graham ID Sullivan K Bruce N . Understanding practice: factors that influence physician hand hygiene compliance. Infect Control Hosp Epidemiol. (2014) 35:1511–20. doi: 10.1086/678597 25419774

[48] Islam R Tinmouth AT Francis JJ Brehaut JC Born J Stockton C . A cross-country comparison of intensive care physicians’ beliefs about their transfusion behaviour: a qualitative study using the theoretical domains framework. Implement Sci. (2012) 7:93. doi: 10.1186/1748-5908-7-93 22999460 PMC3527303

[49] Patey AM Islam R Francis JJ Bryson GL Grimshaw JM The Canada PRIME Plus Team . Anesthesiologists’ and surgeons’ perceptions about routine pre-operative testing in low-risk patients: application of the theoretical domains framework (TDF) to identify factors that influence physicians’ decisions to order pre-operative tests. Implement Sci. (2012) 7:1–13. doi: 10.1186/1748-5908-7-52 PMC3522997 22682612

[50] Saldaña J. The coding manual for qualitative researchers. 2nd Edn. London: SAGE Publications Ltd (2013).

[51] Braun V Clarke V. Using thematic analysis in psychology. Qual Res Psychol. (2006) 3:77–101. doi: 10.1191/1478088706qp063oa

[52] Nzanga M Panulo M Morse T Chidziwisano K. Adherence to hand hygiene among nurses and clinicians at Chiradzulu District hospital, southern Malawi. Int J Environ Res Public Health. (2022) 19:10981. doi: 10.3390/ijerph191710981 36078689 PMC9518139

