# Supplementary material for: Developing an infection prevention and control intervention to reduce hospital-acquired infections in Cambodia and Lao People’s Democratic Republic: the HAI-PC study protocol
Source: Front Public Health. 2023 Sep 20;11:1239228. doi: 10.3389/fpubh.2023.1239228 (PMC10548876; doi:10.3389/fpubh.2023.1239228)
Supplement: Supplementary file 1 [file Data_Sheet_1.docx]

**TOPIC GUIDE FOR KEY INFORMANT INTERVIEWS WITH KEY STAKEHOLDERS**

**To be filled out by the interviewer:**

Date of interview: ______/ _______/ ____________

Interviewer’s name: _________________________

Healthcare facility ID: _______________________________

Unit/Ward ID: ____________________

Time interview started: ________________ Time interview ended: _________________

**Script:**

This study aims to develop and pilot an infection prevention and control (IPC) intervention for reducing hospital-acquired infections (HAIs) in health facilities and assess its feasibility and acceptability in Cambodia and Lao PDR. Through this interview, we would like to explore the feasibility and acceptability of the IPC we have developed and piloted among healthcare workers at national, provincial, and district referral hospitals to identify areas for improvement and support the implementation scale-up.

This research study is anonymous, and participation is voluntary. Upon reading the informed consent, you indicated an interest in participating in the study, and we scheduled this interview. Before starting the interview, we will ask for your written consent. This interview will be conducted in Khmer in Cambodia and Lao in Lao PDR and audio recorded. It is important to note that there are no right or wrong answers to the questions. We will use your recordings for transcription purposes only.

Do you agree to proceed with this interview?

**Start recording:**

Date: ___/____/____

Time: __: __

Interview ID: _____

**Start interview:**

**Questions regarding the participant**

1. How many years have you worked in this healthcare facility/institute/department?
2. How many years of experience do you have in this specific field?
3. What is your usual work profile like?
4. What is your position/role (nurse/midwife/medical doctor)?
5. May I know your age?

**Hospital-acquired infection (HAI) prevention and control**

1. In your opinion, how was this intervention suitable in the Cambodian/Lao context?
   - How was this intervention suitable for healthcare workers?
2. What is the demand to implement this intervention across all hospitals in Cambodia?
3. Do you think this intervention can be successfully delivered to intended healthcare workers?
4. To what extent can healthcare workers carry out this intervention using existing means, resources, and circumstances without outside intervention?
   - In national hospitals
   - In provincial hospitals
   - In district referral hospitals
5. How likely can this intervention be integrated into the existing system?
   - What would be the challenges that you can foresee?
   - Who are the most important people to discuss HAI prevention and control policies?
   - Who are the most important people when you want to obtain HAI prevention and control information?
6. Any other comments/suggestions that you would like to make regarding this IPC intervention?

*This is the end of the interview. Thank you for your time.*
