# Supplementary material for: Developing an infection prevention and control intervention to reduce hospital-acquired infections in Cambodia and Lao People’s Democratic Republic: the HAI-PC study protocol
Source: Front Public Health. 2023 Sep 20;11:1239228. doi: 10.3389/fpubh.2023.1239228 (PMC10548876; doi:10.3389/fpubh.2023.1239228)
Supplement: Supplementary file 2 [file Data_Sheet_2.docx]

HAND HYGIENE OBSERVATION FORM

| **Facility:** |  | **Period Number*****:** |  | **Session Number*****:** |
| --- | --- | --- | --- | --- |
| **Service:** |  | **Date:**  **(dd/mm/yy)** | / / | **Observer:**  **(initials)** |
| **Ward:** |  | **Start/End time:**  **(hh:mm)** | : / : | **Page N°:** |
| **Department:** |  | **Session duration:**  **(mm)** |  | **City******:** |
| **Country******:** |  |  | | |

| **Prof.cat** | |  | | **Prof.cat** | |  | | **Prof.cat** | |  | | **Prof.cat** | |  | |
| --- | --- | --- | --- | --- | --- | --- | --- | --- | --- | --- | --- | --- | --- | --- | --- |
| **Code** | |  | | **Code** | |  | | **Code** | |  | | **Code** | |  | |
| **N°** | |  | | **N°** | |  | | **N°** | |  | | **N°** | |  | |
| **Opp.** | **Indication** | | **HH Action** | **Opp.** | **Indication** | | **HH Action** | **Opp.** | **Indication** | | **HH Action** | **Opp.** | **Indication** | | **HH Action** |
| **1** | bef-pat. bef-asept. aft-b.f.  aft-pat. aft.p.surr. | | HR HW  🌕 missed  🌕 gloves | **1** | bef-pat. bef-asept. aft-b.f.  aft-pat.  aft.p.surr | | HR HW  🌕 missed  🌕 gloves | **1** | bef-pat. bef-asept. aft-b.f.  aft-pat. aft.p.surr. | | HR HW  🌕 missed  🌕 gloves | **1** | bef-pat. bef-asept. aft-b.f.  aft-pat. aft.p.surr. | | HR HW  🌕 missed  🌕 gloves |
|  | | | | | | | | | | | | | | | |
| **2** | bef-pat. bef-asept. aft-b.f.  aft-pat. aft.p.surr. | | HR HW  🌕 missed  🌕 gloves | **2** | bef-pat. bef-asept. aft-b.f.  aft-pat. aft.p.surr. | | HR HW  🌕 missed  🌕 gloves | **2** | bef-pat. bef-asept. aft-b.f.  aft-pat. aft.p.surr. | | HRHW  🌕 missed  🌕 gloves | **2** | bef-pat. bef-asept. aft-b.f.  aft-pat. aft.p.surr. | | HR HW  🌕 missed  🌕 gloves |
|  | | | | | | | | | | | | | | | |
| **3** | bef-pat. bef-asept. aft-b.f.  aft-pat. aft.p.surr. | | HR HW  🌕 missed  🌕 gloves | **3** | bef-pat. bef-asept. aft-b.f.  aft-pat. aft.p.surr. | | HR HW  🌕 missed  🌕 gloves | **3** | bef-pat. bef-asept. aft-b.f.  aft-pat. aft.p.surr. | | HR HW  🌕 missed  🌕 gloves | **3** | bef-pat. bef-asept. aft-b.f.  aft-pat. aft.p.surr. | | HR HW  🌕 missed  🌕 gloves |
|  | | | | | | | | | | | | | | | |
| **4** | bef-pat. bef-asept. aft-b.f.  aft-pat. aft.p.surr. | | HR HW  🌕 missed  🌕 gloves | **4** | bef-pat. bef-asept. aft-b.f.  aft-pat. aft.p.surr. | | HR HW  🌕 missed  🌕 gloves | **4** | bef-pat. bef-asept. aft-b.f.  aft-pat. aft.p.surr. | | HR HW  🌕 missed  🌕 gloves | **4** | bef-pat. bef-asept. aft-b.f.  aft-pat. aft.p.surr. | | HR HW  🌕 missed  🌕 gloves |
|  | | | | | | | | | | | | | | | |
| **5** | bef-pat. bef-asept. aft-b.f.  aft-pat. aft.p.surr. | | HR HW  🌕 missed  🌕 gloves | **5** | bef-pat. bef-asept. aft-b.f.  aft-pat. aft.p.surr. | | HR HW  🌕 missed  🌕 gloves | **5** | bef-pat. bef-asept. aft-b.f.  aft-pat. aft.p.surr. | | HR HW  🌕 missed  🌕 gloves | **5** | bef-pat. bef-asept. aft-b.f.  aft-pat. aft.p.surr. | | HR HW  🌕 missed  🌕 gloves |
|  | | | | | | | | | | | | | | | |
| **6** | bef-pat. bef-asept. aft-b.f.  aft-pat. aft.p.surr. | | HR HW  🌕 missed  🌕 gloves | **6** | bef-pat. bef-asept. aft-b.f.  aft-pat. aft.p.surr. | | HR HW  🌕 missed  🌕 gloves | **6** | bef-pat. bef-asept. aft-b.f.  aft-pat. aft.p.surr. | | HR HW  🌕 missed  🌕 gloves | **6** | bef-pat. bef-asept. aft-b.f.  aft-pat. aft.p.surr. | | HR HW  🌕 missed  🌕 gloves |
|  | | | | | | | | | | | | | | | |
| **7** | bef-pat. bef-asept. aft-b.f.  aft-pat. aft.p.surr. | | HR HW  🌕 missed  🌕 gloves | **7** | bef-pat. bef-asept. aft-b.f.  aft-pat. aft.p.surr. | | HR HW  🌕 missed  🌕 gloves | **7** | bef-pat. bef-asept. aft-b.f.  aft-pat. aft.p.surr. | | HR HW  🌕 missed  🌕 gloves | **7** | bef-pat. bef-asept. aft-b.f.  aft-pat. aft.p.surr. | | HR HW  🌕 missed  🌕 gloves |
|  | | | | | | | | | | | | | | | |
| **8** | bef-pat. bef-asept. aft-b.f.  aft-pat. aft.p.surr. | | HR HW  🌕 missed  🌕 gloves | **8** | bef-pat. bef-asept. aft-b.f.  aft-pat. aft.p.surr. | | HR HW  🌕 missed  🌕 gloves | **8** | bef-pat. bef-asept. aft-b.f.  aft-pat. aft.p.surr. | | HR HW  🌕 missed  🌕 gloves | **8** | bef-pat. bef-asept. aft-b.f.  aft-pat. aft.p.surr. | | HR HW  🌕 missed  🌕 gloves |

* To be completed by the data manager.

** **Optional**, to be used if appropriate, according to the local needs and regulations.
