# Supplementary material for: Developing an infection prevention and control intervention to reduce hospital-acquired infections in Cambodia and Lao People’s Democratic Republic: the HAI-PC study protocol
Source: Front Public Health. 2023 Sep 20;11:1239228. doi: 10.3389/fpubh.2023.1239228 (PMC10548876; doi:10.3389/fpubh.2023.1239228)
Supplement: Supplementary file 3 [file Data_Sheet_3.docx]

**LOW-LEVEL DISINFECTION OF EQUIPMENT OBSERVATION FORM**

**To be filled out by the observer:**

Date of observing: ______/ _______/ ____________

Observer’s name: _________________________

Healthcare facility ID: _______________________________

Unit/Ward ID: ________________________________

Time observation started: ________________ Time observation ended: _________________

| **Equipment** | **Used** | | **If yes, disinfection** | |
| --- | --- | --- | --- | --- |
|  | **No** | **Yes** | **Before use** | **After use** |
| Bedpans |  |  | □ Disinfected with rubbing alcohol/bleach  □ Not disinfected, but cleaned  □ Not disinfected, not cleaned  □ Cannot say | □ Disinfected with rubbing alcohol/bleach  □ Not disinfected, but cleaned  □ Not disinfected, not cleaned  □ Cannot say |
| Blood pressure cuffs |  |  | □ Disinfected with rubbing alcohol/bleach  □ Not disinfected, but cleaned  □ Not disinfected, not cleaned  □ Cannot say | □ Disinfected with rubbing alcohol/bleach  □ Not disinfected, but cleaned  □ Not disinfected, not cleaned  □ Cannot say |
| ECG leads |  |  | □ Disinfected with rubbing alcohol/bleach  □ Not disinfected, but cleaned  □ Not disinfected, not cleaned  □ Cannot say | □ Disinfected with rubbing alcohol/bleach  □ Not disinfected, but cleaned  □ Not disinfected, not cleaned  □ Cannot say |
| Thermometers |  |  | □ Disinfected with rubbing alcohol/bleach  □ Not disinfected, but cleaned  □ Not disinfected, not cleaned  □ Cannot say | □ Disinfected with rubbing alcohol/bleach  □ Not disinfected, but cleaned  □ Not disinfected, not cleaned  □ Cannot say |
| Stethoscopes |  |  | □ Disinfected with rubbing alcohol/bleach  □ Not disinfected, but cleaned  □ Not disinfected, not cleaned  □ Cannot say | □ Disinfected with rubbing alcohol/bleach  □ Not disinfected, but cleaned  □ Not disinfected, not cleaned  □ Cannot say |
| Beds |  |  | □ Disinfected with rubbing alcohol/bleach  □ Not disinfected, but cleaned  □ Not disinfected, not cleaned  □ Cannot say | □ Disinfected with rubbing alcohol/bleach  □ Not disinfected, but cleaned  □ Not disinfected, not cleaned  □ Cannot say |
| Bedside tables |  |  | □ Disinfected with rubbing alcohol/bleach  □ Not disinfected, but cleaned  □ Not disinfected, not cleaned  □ Cannot say | □ Disinfected with rubbing alcohol/bleach  □ Not disinfected, but cleaned  □ Not disinfected, not cleaned  □ Cannot say |
|  |  |  | □ Disinfected with rubbing alcohol/bleach  □ Not disinfected, but cleaned  □ Not disinfected, not cleaned  □ Cannot say | □ Disinfected with rubbing alcohol/bleach  □ Not disinfected, but cleaned  □ Not disinfected, not cleaned  □ Cannot say |
|  |  |  | □ Disinfected with rubbing alcohol/bleach  □ Not disinfected, but cleaned  □ Not disinfected, not cleaned  □ Cannot say | □ Disinfected with rubbing alcohol/bleach  □ Not disinfected, but cleaned  □ Not disinfected, not cleaned  □ Cannot say |

Note: The research team will list equipment during the site visits.

Low-level disinfectants are used to disinfect noncritical items that come into contact with skin. According to the national guidelines on IPC for healthcare facilities, the equipment includes bedpans, toilets, urinals, blood pressure cuffs, electrocardiogram leads, thermometers, stethoscopes, beds, and bedside tables.
