# Supplementary material for: Developing an infection prevention and control intervention to reduce hospital-acquired infections in Cambodia and Lao People’s Democratic Republic: the HAI-PC study protocol
Source: Front Public Health. 2023 Sep 20;11:1239228. doi: 10.3389/fpubh.2023.1239228 (PMC10548876; doi:10.3389/fpubh.2023.1239228)
Supplement: Supplementary file 4 [file Data_Sheet_4.docx]

**TOPIC GUIDE FOR IN-DEPTH INTERVIEWS WITH HEALTHCARE WORKERS**

**To be filled out by the interviewer:**

Date of interview: ______/ _______/ ____________

Interviewer’s name: _________________________

Healthcare facility ID: _______________________________

Unit/Ward ID: ____________________

Time interview started: ________________ Time interview ended: _________________

**Script:**

This study aims to develop and pilot an infection prevention and control (IPC) intervention for reducing hospital-acquired infections (HAIs) in health facilities and assess its feasibility and acceptability in Cambodia and Lao PDR. Through this interview, we would like to explore IPC implementation and practices among healthcare workers at national, provincial, and district referral hospitals to identify barriers and enablers of hand-hygiene practices among healthcare workers for developing IPC intervention components.

This research study is anonymous, and participation is voluntary. Upon reading the informed consent, you indicated an interest in participating in the study, and we scheduled this interview. Before starting the interview, we will ask for your written consent. This interview will be conducted in Khmer in Cambodia and Lao in Lao PDR and audio recorded. It is important to note that there are no right or wrong answers to the questions. We will use your recordings for transcription purposes only.

Do you agree to proceed with this interview?

**Start recording:**

Date: ___/____/____

Time: __: __

Interview ID: _____

**Start interview:**

**Questions regarding the participant**

1. How many months or years have you worked in this healthcare facility? *(Note: eligibility criteria – healthcare workers working in this health facility for at least six months; 18 years and above. Interns and visiting healthcare workers are excluded).*
2. How many months or years of experience do you have in this field?
3. What is your work profile/role (nurse/midwife/doctor, etc.)?
4. May I know your age?

**Hospital-acquired infections (HAIs) prevention and control**

1. What are your views on infection prevention and control in Cambodia/Lao PDR?
2. Are you aware of any policies/guidelines to prevent and control HAIs being implemented in your healthcare facility?
   1. Are there any policies/guidelines specific to your ward?
   2. Can you name some of these policies/guidelines?
   3. So far, have you implemented/applied policies/guidelines?
      - In your opinion, are these policies/guidelines effective? If so, what makes you think it is effective or ineffective?
      - Are guidelines adequate? If so, what makes you think they are adequate or inadequate?
3. What do you think are some of the ways to prevent HAIs in this ward?
   1. Which do you think is the most effective way?
   2. To help me understand further, can you elaborate/explain? What makes you think these are the most effective ways to prevent HAIs?

**Hand-hygiene practices**

1. How are you informed about hand-hygiene policies/guidelines at work?
2. In your opinion, how useful are the current practices in delivering information about the hand-hygiene guidelines in encouraging healthcare workers to follow these guidelines?
   1. Is there information on the five moments for hand hygiene in the guidelines?
   2. Do you intend to practice the five moments for hand hygiene?
3. Have you ever been trained in hand-hygiene techniques using soap and water or alcohol-based hand sanitizer?
   1. When was the last training you had on hand-hygiene practices?
   2. Who conducted the training? Was it sufficient for you to learn?
   3. Did you receive other infection prevention and control (IPC)-related training in the last six months?
   4. Has anyone evaluated your hand-hygiene practices?
4. How important is it for you to practice proper hand hygiene?
5. Are there any changes regarding delivering information about the hand-hygiene guidelines you recommend?

**Effectiveness of hand hygiene**

1. How effective are the hand-hygiene guidelines being implemented in this ward*? [Try asking, “What makes you think that way?”]*
2. Do you think that your colleagues would agree? If yes/no, can you please elaborate?
3. Are there certain situations where you find yourself forgetting to practice hand hygiene?
   1. Is there anything that would make it easier for you to remember to practice hand hygiene?
4. In what situations might you find it difficult to follow the hand-hygiene guidelines?
   1. What would happen if you were not able to practice hand hygiene?
   2. Have you ever experienced difficulties applying proper hand-hygiene practices at work when you wanted to? If so, can you give me a few examples? And in your opinion, how would you address the challenges?
5. In the past, were there any personal or external incentives you have experienced to effectively improve hand hygiene? If so, can you give a few examples? If not, is it something you would like to have?
6. Have you heard others expressing concerns about hand-hygiene practices at your unit/ward? If so, what are the concerns?

**Factors influencing hand-hygiene practices**

1. What aspects of your work environment influence whether you practice hand hygiene?
   1. What resources do you currently have to practice hand hygiene in a recommended way?
   2. Are there any competing tasks or time constraints that influence whether you practice hand hygiene? What would help you overcome these problems/difficulties?
2. To what extent can your colleagues (co-workers/team lead/department/overall workplace) influence your decision to practice good hand hygiene?
3. How do patients and their families influence the practices of hand hygiene?
4. Are there any factors that may motivate you to apply proper hand-hygiene practices? If so, can you please help me understand?
   1. What do you think are the benefits/negative aspects when good hand hygiene is practiced?
   2. What strategies are already in place to support you in hand-hygiene practices? Do you use them? What about your colleagues? Do they follow those practices?
   3. What are additional strategies needed to ensure consistent hand-hygiene practices among your healthcare team?
   4. What could you personally do better to practice good hand hygiene?
5. How does your mood affect you when practicing hand hygiene?
   1. Do you worry if you do not practice hand hygiene?

**Hand-hygiene practices during the COVID-19 pandemic**

1. What impact has the COVID-19 pandemic had on hand-hygiene practices in your ward?
   1. Does the COVID-19 pandemic make it difficult or easy to practice hand hygiene?
   2. And how about the practicality – for example, wearing gloves, workload, accessibility to perform hand hygiene, etc.?
2. How were the hand-hygiene guidelines adapted or changed according to the local COVID-19 epidemiology and the mitigation measures?
3. Have you encountered difficulties in hand-hygiene practices during the COVID-19 pandemic? If so, what are the mechanisms in place to overcome these difficulties?

**Conclusion**

Do you have any other comments, suggestions, or thoughts on hand hygiene or other forms of HAI prevention and control?

*This is the end of the interview. Thank you for your time.*
