# Supplementary material for: Developing an infection prevention and control intervention to reduce hospital-acquired infections in Cambodia and Lao People’s Democratic Republic: the HAI-PC study protocol
Source: Front Public Health. 2023 Sep 20;11:1239228. doi: 10.3389/fpubh.2023.1239228 (PMC10548876; doi:10.3389/fpubh.2023.1239228)
Supplement: Supplementary file 5 [file Data_Sheet_5.docx]

**TOPIC GUIDE FOR KEY INFORMANT INTERVIEWS**

**To be filled out by the interviewer:**

Date of interview: ______/ _______/ ____________

Interviewer’s name: _________________________

Healthcare facility ID: _______________________________

Unit/Ward ID: ____________________

Time interview started: ________________ Time interview ended: _________________

**Script:**

This study aims to develop and pilot an infection prevention and control (IPC) intervention for reducing hospital-acquired infections (HAIs) in health facilities and assess its feasibility and acceptability in Cambodia and Lao PDR. The purpose of this interview is to understand the perspective of key stakeholders about the gaps in infection prevention and control policies, practices, and interventions in the countries.

This research study is anonymous, and participation is voluntary. Upon reading the informed consent, you indicated an interest in participating in the study, and we scheduled this interview. Before starting the interview, we will ask for your written consent. This interview will be conducted in Khmer in Cambodia and Lao in Lao PDR and audio recorded. It is important to note that there are no right or wrong answers to the questions. We will use your recordings for transcription purposes only.

Do you agree to proceed with this interview?

**Start recording:**

Date: ___/____/____

Time: __: __

Interview ID: _____

**Start interview:**

**Questions regarding the participant**

1. How many years have you worked in this healthcare facility/institute/department?
2. How many years of experience do you have in this specific field?
3. What is your work profile/role?
4. Are you a nurse/midwife/medical doctor?
5. May I know your age?

**Hospital-acquired infection (HAI) prevention and control policies and guidelines**

1. What are your views on infection prevention and control (IPC) in Cambodia/Lao PDR?
   - What are your views on the IPC policies/guidelines development in the country?
   - Are there any IPC guidelines introduced by the World Health Organization (WHO) that the Ministry of Health has not adopted?
   - Are you aware of any regional or international IPC consortium your country has or has not been a member of? (For policymakers at the national level)
2. Are you aware of any IPC policies/guidelines in this country being implemented at a national/provincial/district level?
   - What are some of these policies/guidelines?
   - How do relevant health departments or authorities prioritize these policies/guidelines?
   - How effective are these policies/guidelines?
   - How does this policy impact IPC in health facilities?
   - What are the benefits and limitations of these policies/guidelines?
3. In your opinion, who are the target populations for these policies/guidelines?
   - What do you think are their interpretation, attitudes, and practices regarding these policies/guidelines?
   - What is the target population’s opinion regarding these policies/guidelines?
   - What influence do they have to adapt these policies/guidelines?
4. What are your perspectives regarding the following HAI prevention and control assessments? *[Discuss barriers and facilitators here]*
   - Program and infrastructure
   - Training, competency, and implementation of policies and practices
   - Systems to detect, prevent, and respond to HAIs
5. What impact has the COVID-19 pandemic had on HAI prevention and control in this country?
6. How were the HAI prevention and control policies/guidelines adapted or changed according to the local COVID-19 epidemiology and mitigation measures?
7. What are your predictions for HAI prevention in your country (at a national/provincial/district level) based on the current policies, guidelines, and interventions?
   - Where are the gaps?
   - How can the resources be better allocated?
8. What are your recommendations for improving HAI prevention and control policies/guidelines?
   - How can we incorporate the services for better implementation?

**Conclusion**

Do you have any other comments, suggestions, or thoughts on HAI prevention and control?

*This is the end of the interview. Thank you for your time.*
