# Supplementary material for: Developing an infection prevention and control intervention to reduce hospital-acquired infections in Cambodia and Lao People’s Democratic Republic: the HAI-PC study protocol
Source: Front Public Health. 2023 Sep 20;11:1239228. doi: 10.3389/fpubh.2023.1239228 (PMC10548876; doi:10.3389/fpubh.2023.1239228)
Supplement: Supplementary file 6 [file Data_Sheet_6.docx]

**TOPIC GUIDE FOR IN-DEPTH INTERVIEWS AMONG HEALTHCARE WORKERS**

**To be filled out by the interviewer:**

Date of interview: ______/ _______/ ____________

Interviewer’s name: _________________________

Healthcare facility ID: _______________________________

Unit/Ward ID: ____________________

Time interview started: ________________ Time interview ended: _________________

**Script:**

This study aims to develop and pilot an infection prevention and control (IPC) intervention for reducing hospital-acquired infections (HAIs) in health facilities and assess its feasibility and acceptability in Cambodia and Lao PDR. Through this interview, we would like to explore the feasibility and acceptability of the IPC we have developed and piloted among healthcare workers at national, provincial, and district referral hospitals to identify areas for improvement and support the implementation scale-up.

This research study is anonymous, and participation is voluntary. Upon reading the informed consent, you indicated an interest in participating in the study, and we scheduled this interview. Before starting the interview, we will ask for your written consent. This interview will be conducted in Khmer in Cambodia and Lao in Lao PDR and audio recorded. It is important to note that there are no right or wrong answers to the questions. We will use your recordings for transcription purposes only.

Do you agree to proceed with this interview?

**Start recording:**

Date: ___/____/____

Time: __: __

Interview ID: _____

**Start interview:**

**Questions regarding the participant**

1. How many months or years have you worked in this healthcare facility? *(Note: eligibility criteria – healthcare workers working in this health facility for at least six months; 18 years and above. Interns and visiting healthcare workers are excluded).*
2. How many months or years of experience do you have in this field?
3. What is your work profile/role (nurse/midwife/doctor, etc.)?
4. May I know your age?

**Hospital-acquired infection (HAI) prevention and control in Cambodia**

1. In your opinion, how relevant was the intervention in this unit?
   1. What did you expect from this intervention?
   2. Were what you expected from the intervention different from what you received?
2. How successful do you believe the intervention was?
   1. What were the challenges you came across while implementing the intervention?
3. How satisfied are you with the approach we used to develop the intervention?
   1. How did you feel about the contents of the intervention?
   2. How did you feel about the frequency of the intervention?
   3. How did you feel about the delivery time of the intervention?
4. Have you noticed any changes in how you think about hand hygiene and low-level disinfection of equipment as the weeks went by?
   1. What were the changes you observed?
5. What do you think was the most valuable part of the intervention?
6. What do you think was the least valuable part of the intervention?
7. What would be helpful for us to consider for better designing the intervention?
8. Would you recommend the Ministry of Health incorporate this intervention in other hospitals or units?
   1. How practical this intervention would be in the long term?

*This is the end of the interview. Thank you for your time.*
